# Obsessive-compulsive disorder and trichotillomania: a phenomenological comparison

**DOI:** 10.1186/1471-244X-5-2

**Published:** 2005-01-13

**Authors:** Christine Lochner, Soraya Seedat, Pieter L du Toit, Daniel G Nel, Dana JH Niehaus, Robin Sandler, Dan J Stein

**Affiliations:** 1 MRC Unit on Anxiety and Stress Disorders, Department of Psychiatry, University of Stellenbosch, Cape Town, South Africa; 2 Center for Statistical Consultation, Department of Statistics & Actuarial science, University of Stellenbosch, Stellenbosch, South Africa

## Abstract

**Background:**

Similarities between obsessive-compulsive disorder (OCD) and trichotillomania (TTM) have been widely recognized. Nevertheless, there is evidence of important differences between these two disorders. Some authors have conceptualized the disorders as lying on an OCD spectrum of conditions.

**Methods:**

Two hundred and seventy eight OCD patients (n = 278: 148 male; 130 female) and 54 TTM patients (n = 54; 5 male; 49 female) of all ages were interviewed. Female patients were compared on select demographic and clinical variables, including comorbid axis I and II disorders, and temperament/character profiles.

**Results:**

OCD patients reported significantly more lifetime disability, but fewer TTM patients reported response to treatment. OCD patients reported higher comorbidity, more harm avoidance and less novelty seeking, more maladaptive beliefs, and more sexual abuse. OCD and TTM symptoms were equally likely to worsen during menstruation, but OCD onset or worsening was more likely associated with pregnancy/puerperium.

**Conclusions:**

These findings support previous work demonstrating significant differences between OCD and TTM. The classification of TTM as an impulse control disorder is also problematic, and TTM may have more in common with conditions characterized by stereotypical self-injurious symptoms, such as skin-picking. Differences between OCD and TTM may reflect differences in underlying psychobiology, and may necessitate contrasting treatment approaches.

## Background

Trichotillomania (TTM) is characterized by repetitive stereotypical hair-pulling from different sites resulting in noticeable hair loss [1]. Phenomenological observations have suggested that symptoms of repetitive hair-pulling are reminiscent of the compulsions seen in obsessive-compulsive disorder (OCD) [2,3]. For example, both TTM and OCD patients describe compulsive urges and ritualistic behaviours [2,4]. Comorbidity data also suggest some overlap between TTM and OCD [2]. Thus, a number of authors have suggested that TTM might be classified with OCD in a spectrum of disorders having similar phenomenology [4-8].

However, in addition to overlapping phenomenology between OCD and TTM, there are also significant differences. For example, in contrast to compulsions in OCD, hair-pulling in TTM is not in response to obsessive thoughts (such as worry about harm to self or others) but rather because of an irresistible urge and the promise of gratification when pulling out hair [2,6]. Also, unlike patients with OCD whose symptoms change over time in terms of focus and severity (e.g. from washing of hands to checking locks, stoves, appliances, etc) [9], TTM patients usually only present with hair-pulling without evolution to non-self-injurious compulsive rituals.

Examination of demographic variables in OCD and TTM supports the argument that these are two distinctive disorders. TTM is much more prevalent in females (10:1 female to male ratio) [10] whereas OCD is equally common in males and females [11]. Age of onset also differs somewhat: TTM typically presents in early adolescence, with the mean age of onset of hair-pulling in males later than that in females [10,12,13] whereas OCD has its onset from childhood through to early adulthood [14], but with males reporting an earlier onset compared to females [15].

Additional clinical observations further support a distinction between OCD and TTM. Patients with TTM tend to have fewer comorbid obsessive-compulsive symptoms, as well as less depression and anxiety compared to OCD patients [16]. Response prevention in OCD patients eventually leads to anxiety reduction, whereas in people with TTM it may lead to an increase in anxiety [17]. Although a selective response to serotonergic reuptake inhibitors (SRI's) has been suggested to characterize both OCD and TTM, there is good evidence that response to SRI's is sustained in OCD, whereas the evidence-base for the efficacy of these agents in TTM is much more mixed.

Relatively few empirical studies have, however, documented the phenomenological similarities and differences between OCD and TTM [3,18,19]. A large clinical database comprised of patients with OCD and TTM provided us an opportunity to investigate the relationship between these conditions in terms of demographic and clinical variables.

## Methods

### Subjects

Two hundred and seventy eight OCD patients (n = 278: 148 male; 130 female), and 54 TTM patients (n = 54; 5 male; 49 female), ranging in age between 8 and 75 years, took part in the study (Table 1). These patients were referred to our research unit from a wide range of sources (including the OCD Association of South Africa, community based primary care practitioners, and psychiatrists). Either a clinical psychologist or a psychiatrist with expertise in the field interviewed participants. Participants met the Diagnostic and Statistical Manual of Mental Disorders (DSM-IV) criteria [1] for either a primary diagnosis of OCD or TTM on the Structured Clinical Interview for Axis I Disorders (SCID-I) [20]. Patients were included irrespective of whether they were at baseline (i.e. not receiving any form of treatment for their primary psychiatric disorder), or were receiving treatment for OCD / TTM, but those with comorbid OCD and TTM (N = 25) were excluded from subsequent analysis. A history of psychosis was also an exclusion criterion. Referring clinicians were contacted to establish, where possible, a longitudinal expert evaluation of the diagnostic status of the patient. All subjects gave informed written consent to participate after confidentiality was guaranteed and risks and benefits had been fully explained. The study was approved by the Institutional Review Board of the University of Stellenbosch.

**Table 1:** Demographic information: OCD and TTM

**Variables**	**OCD (N = 278)**		**TTM (N = 54)**		**χ^2^**	**P**
Gender	148 male	130 female	5 male	49 female	40.7	<.001
Age (SD)	33.1 (14.4)		31.3 (12.5)			NS
Population group	86.6% Caucasian		79.6% Caucasian			NS
Level of education	50.7% completed high school or higher level education		44.4% completed high school or higher level education			NS
Employment	6.8% unemployed		3.7% unemployed			NS

NS = non-significant

### Interview

Specific demographic data, including age when interviewed, age of onset of OCD/TTM, highest level of education, current employment status, and population group were obtained from all participants. In addition to the SCID-I, and selected parts of the SCID-II (obsessive-compulsive, avoidant, schizotypal, borderline personality disorders) for adult patients (aged 18 or older) [20], the interview also included the Structured Clinical Interview for Obsessive-Compulsive Spectrum Disorders (SCID-OCSD) to determine the presence of other obsessive-compulsive related conditions [21].

The Yale-Brown Obsessive-Compulsive Severity Scale (Y-BOCS) [22] was implemented to assess the severity of OCD symptoms. Severity of hair-pulling symptoms was assessed with the Massachusetts General Hospital Hair-pulling Scale [23]. The Trichotillomania Behaviour Profile (TBP, available from the first author on request) was administered to TTM patients to assess hair-pulling phenomenology.

Patients' level of insight into the senselessness or excessiveness of their symptoms was assessed on the relevant YBOCS item. When an adequate trial of pharmacotherapy with an SRI (i.e. for both OCD and TTM groups, at least 10 weeks on the medication with a minimum of 6 weeks on mid-range dose) had been undertaken, response to pharmacotherapy was assessed using the global improvement item of the Clinical Global Impression (CGI) scale; subjects with CGI scores of 1 ('very much improved') or 2 ('much improved') were defined as responders [24]. Similarly, when patients received an adequate trial of cognitive behavioural therapy (CBT) (i.e. for both OCD and TTM groups, 8 or more sessions with an expert CBT psychotherapist), response to treatment was rated using the CGI. The Disability Profile questionnaire (DP) [25] was included in the interview to assess current (i.e. past two weeks) and lifetime impairment in eight domains. The DP was initially developed for use in patients with social anxiety disorder; nevertheless, the scale has since been used to assess disability in patients with other anxiety disorders as well [26].

Questions addressing potential precipitating or exacerbating factors, including the impact of menstrual/reproductive cycle changes, brain trauma and history of autoimmune infections on OCD/TTM symptom fluctuations, were included in the interview.

### Self-report questionnaires

Severity of comorbid depression was evaluated with the Beck Depression Inventory (BDI) [27]. The Childhood Trauma Questionnaire (CTQ) [28], a scale proven to be a valid and reliable measure of past traumatic experiences [29], was used as a self-report questionnaire to assess the nature and severity of childhood trauma. Sub-scales of the CTQ include measures of emotional abuse, physical abuse, sexual abuse, emotional neglect and physical neglect.

The self-report Temperament and Character Inventory (TCI) [30] was also used to measure behaviours associated with seven personality dimensions, namely novelty seeking, harm avoidance, reward dependence, persistence, self-directedness, cooperativeness, and self-transcendence. In addition, participants completed the self-report Young Schema Questionnaire (YSQ) [31] to assess the current profile of fundamental maladaptive beliefs (cognitive schemas) in OCD and TTM. For each item of the 75-item "short form" of the YSQ (which includes 15 schemas), the answer is required to be placed on a 6-point Likert-type scale (1= 'completely untrue of me', 2 = 'mostly untrue of me', 3 = 'slightly more true than untrue', 4 = 'moderately true of me', 5 = 'mostly true of me', 6 = 'describes me perfectly').

### Data analysis

As there were few males with TTM (Table 2), only clinical data from females with OCD and TTM were analyzed. Chi-square and t-tests were performed to investigate the differences in OCD/TTM phenomenology where appropriate. A one-way analysis of variance (ANOVA) was done to investigate the effects of the primary and comorbid disorders on disability. Subsequently, a two-way fixed effects ANOVA was used to assess the main interactions between primary diagnosis and comorbidity on disability and to test for the main (fixed) effects of the different diagnoses on disability. Residuals of ANOVA's of cognitive schema data in OCD and TTM groups suggested non-normality of the data. As a result, pair-wise comparison tests (Mann-Whitney U) were implemented to compare the two groups on cognitive schemas.

**Table 2:** Comparison of symptomatology: OCD *vs *TTM

**Variables**	**OCD**	**TTM**	**P**
Age of onset (SD)	19.3 (12.0)	11.8 (7.6)	<.001
Symptom severity (SD)	YBOCS score: 20.1 (8.0); range: 0 – 39	MGHHPS score: 16.1 (6.5); range: 0 – 26	
Severity of depressive symptoms (BDI score)	8.9 (11.3)	5.5 (7.2)	.04
Poor insight	13.6%	0%	NS
Treatment response	SRI: 90.7%	SRI: 42.9%	.003
	CBT: 73.3%	CBT: 33.3%	.02
Tics	12.3%	6.1%	NS

## Results

### Demographics

Gender distribution of the sample differed significantly, with a marked predominance of female participants with TTM compared to almost equal numbers of male and female participants with OCD. TTM patients had an earlier age of onset of illness compared to patients with OCD (Table 2).

### Clinical features

#### Comorbidity

A number of disorders were more frequent in females with OCD: major depressive disorder (MDD), dysthymia, panic disorder, hypochondriasis and intermittent explosive disorder. In terms of the selected Axis II disorders, obsessive-compulsive personality disorder (OCPD) was more frequent in females with OCD (Table 3).

**Table 3:** Lifetime comorbidity: OCD *vs *TTM

**Disorder**	**OCD (N = 130)**	**TTM (N = 49)**	**χ^2^**	**P**
Major depressive disorder	66.9%	49.0%	4.8	.03*
Dysthymia	13.8%	2.0%	6.8	.009**
Bipolar disorder	0.8%	0%	0.6	0.4
Panic disorder	20.8%	6.1%	6.4	.01*
Alcohol abuse	5.4%	2.0%	1.1	0.3
Alcohol dependence	0.8%	0%	0.6	0.4
Substance abuse	0.8%	4.1%	2.0	0.2
Substance dependence	1.5%	2.0%	0.1	0.8
Social phobia	10.0%	8.2%	0.1	0.7
Specific phobia	18.5%	18.4%	0.0	<1.0
Posttraumatic stress disorder	3.1%	0%	2.6	0.1
Generalized anxiety disorder	13.1%	20.4%	1.4	0.2
Body dysmorphic disorder	6.2%	6.1%	0.0	<1.0
Anorexia Nervosa	8.5%	2.0%	2.9	0.1
Bulimia Nervosa	7.7%	6.1%	0.1	0.7
Binge-eating disorder	4.6%	10.2%	1.8	0.2
Hypochondriasis	4.6%	0%	3.9	<.05*
Stereotypic movement disorder	3.5% (N = 85)	0% (N = 18)	1.2	0.3
Tourette's disorder	3.8%	2%	0.4	0.5
Tics	12.3%	6.1%	1.6	0.2
Kleptomania	4.6%	4.1%	0.0	0.9
Pyromania	0%	2.0%	2.6	0.1
Compulsive shopping	6.9%	4.1%	0.5	0.5
Hypersexual disorder	1.5%	0%	1.3	0.3
Intermittent explosive disorder	16.2%	6.1%	3.5	.06
OCPD	39.2%	13.3%	11.3	.001**
Avoidant personality disorder	21.2% (N = 99)	0.03% (N = 26)	2.9	0.9
Schizotypal personality disorder	5.3% (N = 94)	0% (N = 25)	2.4	0.1
Borderline personality disorder	22.3% (N = 94)	8% (N = 49)	3.0	0.1

* p < .05 (2-tailed)

** p < .01 (2-tailed)

#### Symptom severity

The severity of OC symptoms in OCD patients, as measured by the YBOCS severity scale, was 20.1 (± 8.0). TTM patients scored 16.1 (± 6.5) on average on the MGHHPS. Compared to TTM patients, females with OCD had significantly higher depressive symptom scores on the BDI (Table 2).

#### Disability

The DP was administered to a total of 95 OCD and 30 TTM patients (Table 4). OCD patients reported significantly more lifetime impairment due to their illness than TTM patients. More specifically, OCD patients were more impaired in terms of work-related functioning, family functioning, marriage / dating, activities of daily life, and other activities (which included religious activities, membership of clubs, having hobbies, participation in sports etc.) and had more suicidality. One-way ANOVA's showed that the primary diagnosis (either OCD or TTM) (F = 11.84; p = 0.001) and panic disorder (F = 6.73; p = 0.01) had a significant effect on the levels of disability. However, there was no significant interaction effect between the primary diagnosis and panic disorder, suggesting that the levels of disability were dependent on primary diagnosis (either OCD or TTM) (F = 5.79; p = 0.02) and not influenced by the absence or presence of panic disorder (F = 0.001; p = 0.98).

**Table 4:** Disability profile: OCD *vs *TTM

**DOMAIN**	**OCD (N = 95)**			**TTM (N = 30)**			**Mann-Whitney U**	
	
	**Median**	**Min**	**Max**	**Median**	**Min**	**Max**	**Z**	**P**
School	1.0	0	4.0	1.0	0	4.0	-1.2	NS
Work	2.0	0	4.0	1.0	0	3.0	3.7	<0.001
Family	2.0	0	4.0	1.0	0	4.0	2.4	0.02
Marriage / dating	2.0	0	4.0	1.0	0	4.0	3.1	0.002
Friendships	1.0	0	4.0	1.0	0	3.0	1.1	NS
Other activities	2.0	0	4.0	0	0	3.0	2.3	0.02
Activities of daily life	2.0	0	4.0	0	0	3.0	5.5	<0.001
Suicide	1.0	0	4.0	0	0	3.0	2.6	0.009
Total disability	12.0	1.0	26.0	6.5	0	18.0	3.3	<0.001

#### Character / Temperament

Compared to OCD patients, patients with TTM scored significantly higher on novelty seeking, whereas OCD patients had significantly greater harm avoidance (Table 5).

**Table 5:** Temperament and Character Inventory: OCD *vs *TTM

TEMPERAMENT / CHARACTER TRAITS*	**OCD (N = 68)**	**TTM (N = 21)**	**F**	**P**
NS	17.6 (6.8)	21.6 (6.0)	.5	.02
HA	22.3 (7.9)	15.8 (7.3)	.4	.001
RD	22.3 (4.2)	21.8 (4.9)	.4	NS
SD	25.1 (8.2)	28.2 (9.6)	.7	NS
C	30 (5.1)	29.3 (6.8)	2.8	NS
ST	15.2 (6.7)	21.3 (18.3)	5.0	NS

* NS = novelty seeking total score SD = self-directedness total score

HA = harm avoidance total score C = cooperativeness total score

RD = reward dependence total score ST = self-transcendence total score

#### Schemas

Fifty-nine OCD and 26 TTM patients fully completed the YSQ. Pair-wise comparison tests (Mann-Whitney U) indicated that OCD and TTM patients differed significantly on 5 schemas, i.e. mistrust / abuse, social isolation, shame / defectiveness, subjugation and emotional inhibition (Table 7). More specifically, OCD patients had significantly higher scores on each of these schemas compared to TTM patients.

**Table 7:** OCD and TTM scores on the YSQ subscales

**Schemas**	**OCD (n = 59)**			**TTM (n = 26)**			**Mann-Whitney U**	
	Median	Min	Max	Median	Min	Max	Z	P

Emotional deprivation	2.4	1.0	5.6	2.3	1.0	5.4	-0.6	NS
Abandonment	2.8	1.0	6.0	2.1	1.2	6.0	-0.5	NS
Mistrust / abuse	2.6	1.0	5.8	1.9	1.9	5.4	-2.3	.02
Social isolation	2.4	1.0	6.0	1.9	1.9	6.0	-2.7	.007
Shame / defectiveness	2.2	1.0	6.0	1.4	1.4	4.8	-3.0	.003
Failure to achieve	2.0	1.0	6.0	1.9	1.9	4.8	-0.9	NS
Incompetence	2.0	1.0	4.8	1.8	1.8	4.4	-1.2	NS
Vulnerability to harm	2.2	1.0	6.0	1.6	1.6	5.2	-1.8	NS
Enmeshment	1.8	1.0	6.0	1.7	1.7	4.2	-0.6	NS
Subjugation	2.2	1.0	6.0	1.7	1.7	5.6	-2.8	.005
Self-sacrifice	3.4	1.2	6.0	3.2	3.2	5.8	-1.0	NS
Emotional inhibition	2.4	1.0	5.0	1.4	1.4	4.4	-3.8	<.001
Unrelenting standards	3.8	1.2	6.0	3.6	3.6	6.0	-0.9	NS
Entitlement	2.6	1.0	5.8	2.7	2.7	6.0	-0.7	NS
Self-discipline	3.0	1.2	6.0	2.8	2.8	4.8	-1.3	NS

### Precipitating factors

#### Interpersonal trauma history

OCD patients reported more childhood sexual abuse than did TTM patients (p = .04).

#### Brain trauma history

OCD and TTM patients did not differ significantly in terms of a history of serious head injury associated with the onset of OCD or TTM.

#### History of autoimmune infections

Compared to none in the TTM group, 9 OCD patients reported onset of their OCD with an episode of bacterial pharyngitis (p = .06). In terms of other autoimmune infections, OCD and TTM patients did not differ significantly.

#### Hormonal influence

Female OCD and TTM patients did not differ significantly in terms of the impact of premenstrual/menstrual/menopausal symptoms on their illness. Compared to 42 (38.5%) of 109 OCD patients who reported OC symptom changes in the premenstrual/menstrual period, 16 of 32 TTM patients (50%) reported regular changes in their symptoms during this time. Seventeen (n = 17) OCD patients were menopausal and 35.3% (n = 6) of these women reported that their OC symptoms only started with menopause. One of the TTM patients had gone through menopause with no effect on her hair-pulling symptoms. However, OCD and TTM patients differed significantly in terms of the temporal association between pregnancy/puerperium and onset of illness: 42.6% (26 of 61) OCD patients reported OCD onset while pregnant or within a month of childbirth, compared to 7.7% (1 of 13) of TTM patients (χ^2 ^= 6.8; p = .009).

### Treatment response

Significantly fewer TTM patients reported a clinical response to either CBT- or SRI-treatment than did OCD patients (Table 2).

## Discussion

A comparison of women with TTM and with OCD found significant differences in clinical variables; OCD patients had more comorbidity, greater disability, increased childhood interpersonal trauma (specifically sexual abuse) and more maladaptive schemas. Fewer TTM patients, however, reported having responded to treatment.

The gender ratio findings in both OCD and TTM groups were similar to other surveys where a mean female:male ratio of 1.5:1.0 in OCD [11,32,33] and approaching 10:1 in TTM [34] were documented. OCD patients' mean total score on the YBOCS (i.e. 20.1 ± 8.0) puts them in the "moderate" severity category [22]. The mean hair-pulling severity score on the MGHHPS (16.1 ± 6.5) was similar to that reported in other studies [35,36]. Taken together, these data suggest that our patients are not dissimilar from those assessed at other sites.

Our comorbidity findings are consistent with existing data suggesting that depressive and anxiety disorders are highly prevalent in both OCD and TTM, and significantly more prevalent in OCD [3,16,18]. Indeed, compared to TTM, comorbidity in OCD is greater across a range of different diagnostic categories including mood (MDD, dysthymia), anxiety (panic disorder), OCD-related (hypochondriasis) and personality disorders (OCPD). Such comorbidity appears to extend also to impulse control disorders (intermittent explosive disorder), a finding which argues against the current classification of trichotillomania as a member of this spectrum of conditions.

Our findings of increased disability in OCD is consistent with studies on OCD suggesting it is one of the most impairing of all medical disorders [37]. A number of clinical studies have emphasized the burden of OCD across different domains, including higher rates of divorce and separation than in subjects without OCD [13] and significantly impaired instrumental functioning (work, school, home making and family life) [38-41]. However, the impairment and distress due to TTM should not be underestimated: TTM can be associated with serious sociological and psychological effects (e.g. strong feelings of shame and embarrassment [42], as well as avoidance behaviour including potentially dangerous avoidance of medical care [43]) resulting in a significant decline in quality of life (QOL) for patients, their family members and significant others [44,45].

OCD patients reported significantly more sexual abuse than TTM patients (p = .04). This finding differs from our previous data suggesting similar rates of childhood interpersonal trauma (CIT) in OCD and TTM [46]. However, the current sample size is much increased, resulting in more power to detect smaller differences. Indeed, increased rates of OCD (and other anxiety disorders) have previously been linked with a history of physical and sexual abuse during childhood [47,48]. Nevertheless, in both OCD and TTM, dissociative symptoms – which are present in a minority of patients in both conditions – are positively correlated with a history of childhood interpersonal trauma [49], so that a potential role for CIT in some TTM patients should not be ignored [50].

TTM patients had significantly more novelty seeking (NS) than OCD patients, whereas OCD patients scored significantly higher on harm avoidance (HA) compared to TTM. Our findings are consistent with previous work on temperament / character in OCD, showing increased HA and decreased NS [51-53]. Of note, compared with mean temperament scores obtained in a normal community sample [30], both TTM and OCD scored high on HA. NS scores in the TTM sample were higher than in the OCD sample, but compared to normal controls, these fell in the "medium" range. The higher NS in TTM may however point to greater dopaminergic involvement in this disorder, and might also be used to argue that TTM lies closer to the more impulsive risk-/novelty-seeking pole of an impulsive-compulsive (IC) spectrum of disorders [54].

OCD patients had more maladaptive cognitive schemas than TTM, i.e. mistrust / abuse, social isolation, shame / defectiveness, subjugation and emotional inhibition. The schemas that OCD and TTM patients differed on are included in 2 of the 4 higher order factors (i.e. "impaired autonomy" and "disconnection") described by Lee and colleagues' YSQ factor model [55]. While schemas are thought to represent responses to life experience, including the experience of a disorder, they may also reflect underlying symptoms. Given that maladaptive schemas in OCD were not reminiscent of its characteristic symptoms, it is likely that they at least partly reflect life experience. An increased number of maladaptive schemas in OCD is consistent with higher rates of comorbidity, disability, and functional impairment. Nevertheless, further empirical investigation is needed to assess the relationship between schemas and illness course.

Hormonal influences have previously been investigated in OCD [56] and TTM [57]. For example, it has been noted that menarche, premenstruum, pregnancy [58], and menopause [59] may be related to onset or relapse in OCD. Similarly, in a study that investigated the relationship of the menstrual cycle and pregnancy to compulsive hair-pulling, premenstrual symptom exacerbation was reported for actual hair-pulling, urge intensity and frequency, and ability to control pulling [57]. In that study the impact of pregnancy on TTM was less clear. Our findings suggest that significantly more OCD patients than TTM patients report an association between pregnancy/puerperium and the onset of illness. This finding is in part consistent with previous work suggesting that the postpartum may constitute a risk for the onset of OCD in women [60]. Taken together our data suggest both similarities and differences in the role of sexual hormones in the mediation of OCD and TTM.

Although rare, brain injury may play a role in some cases of OCD [61,62]; in only one OCD patient (and none of the TTM patients), head injury was associated with onset of obsessive-compulsive symptoms. No data could be found on the potential role of brain injury in the etiology of hair-pulling.

A number of patients associated the onset of their OCD onset with an infection, possibly bacterial pharyngitis. This finding is consistent with a body of data suggesting post-streptococcal disease is a cause of OCD in children and adolescents [63], and perhaps also adults [64,65]. There is less work demonstrating a role for autoimmune factors in TTM [66]. Notably, the data on bacterial pharyngitis were based on retrospective assessment and could have been contaminated by memory bias.

In our study, more OCD patients reported a positive response to treatment (with CBT or SRI's) than TTM patients. These data should be interpreted cautiously given the retrospective assessments. Nevertheless, there is evidence that SRI's in TTM may not be as effective over the long-term as in OCD [2]. About 40–60% of OCD patients respond to the first trial of an SRI [67], with a proportion of non-responders to a single SRI responding to administration of a second SRI [68]. In comparison, it has been suggested that TTM patients judge their treatment (including pharmacotherapy, psychotherapy, and behaviour modification) to be relatively ineffective [69]. The usefulness of SRI's in TTM has been investigated in a number of studies with results so far being equivocal. For example, Christenson et al [70] were unable to document efficacy for fluoxetine in a placebo-controlled trial in which patients received 6 weeks of the active agent in doses of up to 80 mg/day. Anecdotal evidence also suggests that the effectiveness of SRI's in TTM may wane with time [71].

Although there is evidence for the usefulness of behaviour therapy in both OCD [72] and TTM [73], the focus of the treatment differs in the two disorders (exposure and response prevention in OCD versus habit reversal in TTM). Keuthen et al [74] have suggested that "state-of-the-art" behavioral and pharmacological treatments offer substantial clinical benefit to patients with TTM, but in general clinics there may be relatively little experience with highly specialized interventions.

Several limitations of the current study should be acknowledged. First, interviewers were not blind to the patients' psychiatric diagnosis, so potentially biasing clinician's assessments. Nevertheless, a structured diagnostic instrument ensures a reasonable degree of reliability. Second, instruments employed in the current study are intended for use in adults rather than younger subjects. However, in the case of children and adolescents, the SCID-I was supplemented with a clinical interview of parents or guardians, and self-report data was included only when it was clear that questionnaires had been completed meaningfully. Third, source of referral, and the duration of OCD/TTM, were not controlled for in the analysis, so potentially biasing the analyses. However, given the chronicity of both conditions, this is unlikely to have materially affected the findings. Fourth, males, as well as patients with comorbid OCD and TTM were excluded from the investigation; so that the results here may not be generalizable to all OCD or TTM subjects. Given evidence that the phenotype of OCD [75] and of TTM [10] varies with gender, additional work on male subjects should be undertaken in the future.

## Conclusions

In conclusion, our data suggest that despite some overlap, TTM differs from OCD in terms of demographics (gender distribution), associated clinical variables (e.g. comorbidity, cognitive schemas, temperament/character profiles and disability), precipitating factors (trauma history) and treatment response. It has been suggested that although TTM is not the same as OCD, it lies on a compulsive-impulsive spectrum of disorders [54]. However, it is notable that impulsivity may be an important component of OCD [76], and rather than viewing OCD and TTM on a single dimension, compulsivity and impulsivity should arguably therefore be seen as lying on orthogonal dimensions. Although TTM patients had more novelty seeking, OCD patients were more likely to have intermittent explosive disorder; such data support a view that TTM should not be classified as an impulse control disorder. Indeed, TTM may have more in common with conditions characterized by stereotypical self-injurious symptoms, such as skin-picking [77]. Differences between OCD and TTM may reflect contrasts in underlying psychobiology, and may necessitate contrasting treatment approaches.

## Competing interests

The author(s) declare that they have no competing interests.

## Authors' contributions

CL:

• participated in the design of the study,

• was responsible for patient recruitment,

• did most of the clinical assessments,

• performed the statistical analyses,

• helped to obtain funds for the research, and

• was responsible for the final writing up of data.

SS:

• participated in the design of the study,

• assisted with recruitment of patients, and

• supervised writing and statistical analyses.

PLduT:

• participated in the design of the study,

• assisted with recruitment of patients, and

• did some of the clinical assessments.

DGN:

• was the primary statistical consultant for analyses.

DJHN:

• participated in the design and coordination of the study,

• was responsible for patient recruitment, and

• assisted with clinical assessments.

RS:

• assisted with literature review of cognitive schema data, and

• asisted statistical analysis of schema data.

DJS:

• conceived of the study

• supervised coordination, statistical analysis, and writing,

• did the final revision of paper before submission, and

• assisted with obtaining of funds.

All authors read and approved the final manuscript.

**Table 6:** TCI-temperament cut-off scores: A normal community sample*

**TCI – TEMPERAMENT TRAITS**					
**NS**			**HA**		

LOW	MEDIUM	HIGH	LOW	MEDIUM	HIGH
16	19.5	22	8	12.5	16

*from Cloninger et al, 1994 (reference nr. 31)

## Pre-publication history

The pre-publication history for this paper can be accessed here:


